# CSF3R, SETBP1 and CALR mutations in chronic neutrophilic leukemia

**DOI:** 10.1186/s13045-014-0077-1

**Published:** 2014-10-15

**Authors:** Yajuan Cui, Bing Li, Robert Peter Gale, Qian Jiang, Zefeng Xu, Tiejun Qin, Peihong Zhang, Yue Zhang, Zhijian Xiao

**Affiliations:** MDS and MPN Centre, Institute of Hematology and Blood Diseases Hospital, Chinese Academy of Medical Sciences & Peking Union Medical College, 288 Nanjing Road, Tianjin, 300020 China; Hematology Research Centre, Division of Experimental Medicine, Department of Medicine, Imperial College, London, UK; Peking University People’s Hospital, Peking University Institute of Hematology, Beijing, China; Department of Pathology, Institute of Hematology and Blood Diseases Hospital, Chinese Academy of Medical Sciences & Peking Union Medical College, Tianjin, China; State Key Laboratory of Experimental Hematology, Institute of Hematology and Blood Diseases Hospital, Chinese Academy of Medical Sciences & Peking Union Medical College, Tianjin, China

**Keywords:** Chronic neutrophilic leukemia, CNL, CSF3R, SETBP1, CALR, Mutation

## Abstract

The WHO 2008 definition of chronic neutrophilic leukemia (CNL) is based on clinical and laboratory parameters but not on molecular abnormalities. Mutations in *CSF3R, SETBP1* and *CALR* are reported in patients with chronic neutrophilic leukemia (CNL). However, because CNL is rare, there are few large studies of this issue. We sequenced these genes in 14 patients who met the WHO-criteria of CNL. 8 subjects had *CSF3R*^*T618I*^, 6 *SETBP1* mutations and 1 a *CALR* mutation. Our data suggest mutation analysis of *CSF3R, SETBP1* and *CALR* should be included in the diagnostic criteria for CNL. These data may also have therapy implications.

## To the Editor

The WHO defines chronic neutrophilic leukemia (CNL) as a myeloproliferative neoplasm (MPN) with sustained elevated neutrophils and <10% immature cells [1]. Recently, recurrent somatic mutations in the membrane proximal domain of *CSF3R* were reported in patients with CNL [2,3]. CSF3R was mutated in 100% [3], *SETBP1* 33% [3] and *CALR* in 12.5% of WHO-defined cases of CNL [4]. We analyzed mutations in *CSF3R, SETBP1* and *CALR* in 14 subjects who met the WHO-criteria.

## Findings

*CSF3R* exon 14–17 [3], *SETBP1* exon 4 [3] and *CALR* exon 9 [5] were amplified by PCR and sequenced. 8 subjects who met the WHO 2008 CNL criteria had a *CSF3R*^T618I^ mutation, 6 *SETBP1* mutations (2 D868N, 2 I871T, 1 G870S and 1 D874N) and the last had a *CALR* mutation (c.1154-1155insTTGTC). All mutations were heterozygous except 1 case of *SETBP1*^I871T^. 6 other subjects, 2 with monoclonal gammopathy of unknown significance (MGUS)-associated CNL and 4 with reactive neutrophilic leukocytosis had no mutation of these genes. No subject had a JAK2^V617F^ mutation (Table 1).

**Table 1:** **Clinical characteristics and laboratory variables**

**No.**	**Diagnosis**	**Gender/Age**	**Hb (g/L)**	**WBC (×10** ^**9**^ **/L)**	**ANC (×10** ^**9**^ **/L)**	**PLT (×10** ^**9**^ **/L)**	**Spleen (cm,LCM)**	**Karyotypes**	**CSF3R**	**SETBP1**	**CALR**	**JAK2** ^**V617F**^	**Treatment**	**Survival (month)**
1	CNL	M/80	120	27.19	25.42	91	3	46,XY,t(1,7) (p32,q11)[10]	T618I	D874N	wt	wt	Hydroxyurea	52
2	CNL	M/64	123	86.83	ND	394	0	46,XY[5]	T618I	D868N	wt	wt	Hydroxyurea	5+
3	CNL	F/77	125	35.68	25.92	351	4	46,XY[20]	T618I	G870S	wt	wt	Hydroxyurea	22+
4	CNL	F/49	104	85.61	79.47	20	6	46,XX[20]	T618I	I871T	wt	wt	Hydroxyurea	13+
5	CNL	M/70	86	146.77	121.82	104	15	ND	T618I	I871T	wt	wt	Hydroxyurea	17
6	CNL	M/43	55	112.65	101.1	98	10	46,XY[5]	T618I	D868N	wt	wt	Hydroxyurea	4+
7	CNL	F/69	102	57.40	40.16	231	6	46,XX[12]	T618I	wt	c.1154-1155insTTGTC	wt	Hydroxyurea	10
8	CNL	M/45	119	32.76	28.73	286	6	46,XY[9]	T618I	wt	wt	wt	Hydroxyurea	32
9	MGUS-CNL	M/46	63	65.30	60.7	101	7	46,XY[2]	wt	wt	wt	wt	Hydroxyurea	6+
10	MGUS-CNL	F/52	121	26.53	19.94	170	3	46,XX[20]	wt	wt	wt	wt	Hydroxyurea	27+

All the parameters in Table 1 were measured at the initial diagnosis in our hospital.

MGUS-CNL: monoclonal gammopathy with uncertain significance associated CNL; Hb: hemoglobin; WBC: White Blood Cell Count; ANC: Absolute Neutrophil Count; PLT: Platelet Count; Spleen (cm): Spleen size under left costa. ND: not done.

The consistent association between CSF3RT618I and CNL in our study is similar to data of Maxson *et al*. [2] and Pardanani *et al*. [3] (Table 2). Tefferi *et al.* [6] suggested including *CSF3R*^T618I^ or other membrane proximal *CSF3R* mutations as a criteria for diagnosis of CNL. We also confirmed the high incidence *SETBP1* mutations in patients with CNL. The mutations we detected focused on a hotspot area from D868 to D874 (Table 1). Although these mutations also occur in other hematologic neoplasms such as atypical chronic myeloid leukemia aCML and chronic myelomonocytic leukemia (CMML), analysis of *SETBP1* mutations could help distinguish CNL from reactive conditions such as infection, inflammatory conditions and non-haematologic neoplasms.

**Table 2:** **CSF3R and SETBP1 mutations in CNL**

	**Gotlib J et al. (2013) [** 7 **]**	**Pardanani A et al. (2013) [** 3 **]**	**This series**	**Total**
T618I only	1	5	1	7
T618I + SETBP1	4	4	6	14
Compound CSF3R mutations^a^ only	2	1	0	3
Compound CSF3R mutations + SETBP1 mutations	1	0	0	1
Others	1^b^	2^c^	1^d^	4

^a^: compound CSF3R mutations mean nonsense or frameshift mutations that truncate the cytoplasmic tail (truncation mutations) combined with point mutations in the extracellular domain (membrane proximal mutation). In the 3 cases of compound CSF3R mutations Tyner et al. reported, two patients harbored T618I and one harbored T615A in the membrane proximal domain. In Tefferi’s study, the compound CSF3R mutation showed T618I + c.2341_2342insC.

^b^: A case with JAK2 mutation only.

^c^: A case with I598I and a case with M696T in CSF3R.

^d^: A case with CSF3R T618I and CALR frameshift mutation.

Gotlib *et al.* reported JAK2^V617F^ mutation in a subject of CNL [7]. Lasho *et al.* reported a *CALR* mis-sense mutation in a subject with CNL [4]. We found concurrent *CSF3R*^T618I^ and *CALR* frame-shift mutations in 1 subject. The 5 bp insertion into *CALR* exon 9 is reported in *BCR/ABL1-* and *JAK2*-negative MPNs and results in a 1+ base-pair frame-shift with an altered C-terminus.

There is controversy whether co-existence of MGUS and CNL is one or two diseases. The 2 MGUS subjects in our study had no mutation in *CSF3R, SETBP1, JAK2* or *CALR*. In another study, none of 6 cases of MGUS-associated CNL had *CSF3R* mutations [3]. Also, survival of patients with MGUS-associated CNL is significantly longer survival than those with CNL only. These data support the notion patients with MGUS and CNL are 2 diseases [8].

There may be therapy implications of our findings. *CSF3R* truncation mutations may be sensitive to SRC kinase-inhibitors such as dasatinib whereas *CSF3R* membrane proximal mutations may be sensitive to JAK kinase-inhibitors such as ruxolitinib [9,10]. Ruxolitinib was reportedly effective in a mouse model of CNL and a patient with CNL and a *CSF3R*^T618I^ mutation [2,11]. However, ruxolitinib was ineffective in a patient with *CSF3R*^T618I^ and *SETBP1* mutations in whom fedratinib suppressed CFU-GM colony formation [12].
